# MicroRNA Expression Profiles in Autism Spectrum Disorder: Role for miR-181 in Immunomodulation

**DOI:** 10.3390/jpm11090922

**Published:** 2021-09-17

**Authors:** Richard E. Frye, Shannon Rose, Sandra McCullough, Sirish C. Bennuri, Patricia A. Porter-Gill, Harsh Dweep, Pritmohinder S. Gill

**Affiliations:** 1 Barrow Neurological Institute at Phoenix Children’s Hospital, Phoenix, AZ 85016, USA; 2 Department of Child Health, University of Arizona College of Medicine, Phoenix, AZ 85004, USA; 3 Department of Pediatrics, University of Arkansas for Medical Sciences, Little Rock, AR 72202, USA; SROSE@UAMS.EDU (S.R.); PSGill@uams.edu (P.S.G.); 4 Arkansas Children’s Research Institute, Little Rock, AR 72202, USA; McCulloughSandraS@uams.edu (S.M.); SCBennuri@uams.edu (S.C.B.); PortergillPA@archildrens.org (P.A.P.-G.); 5 The Wistar Institute, 3601 Spruce St, Philadelphia, PA 19104, USA; hdweep@Wistar.org

**Keywords:** AKT2, AKT3, autism spectrum disorder, CamKinase II, microRNA, miR-181, sibling study, TNF alpha

## Abstract

Background: MicroRNAs (miRNAs) are important regulators of molecular pathways in psychiatric disease. Here, we examine differential miRNAs expression in lymphoblastoid cell lines (LCLs) derived from 10 individuals with autism spectrum disorder (ASD) and compare them to seven typically developing unrelated age- and gender-matched controls and 10 typically developing siblings. Small RNAseq analysis identified miRNAs, and selected miRNAs were validated using quantitative real-time polymerase reaction (qRT-PCR). KEGG analysis identified target pathways, and selected predicted mRNAs were validated using qRT-PCR. Results: Small RNAseq analysis identified that multiple miRNAs differentiated ASD from unrelated controls and ASD from typically developing siblings, with only one, hsa-miR-451a_R-1, being in common. Verification with qRT-PCR showed that miR-320a differentiated ASD from both sibling and unrelated controls and that several members of the miR-181 family differentiated ASD from unrelated controls. Differential expression of AKT2, AKT3, TNF α and CamKinase II predicted by KEGG analysis was verified by qRT-PCR. Expression of CamKinase II βwas found to be correlated with the severity of stereotyped behavior of the ASD participants. Conclusions: This study provides insight into the mechanisms regulating molecular pathways in individuals with ASD and identifies differentiated regulated genes involved in both the central nervous system and the immune system.

## 1. Introduction

Autism spectrum disorder (ASD) is a complex neurodevelopmental condition defined by abnormalities in social communication along with restricted and repetitive behaviors and interests [1]. ASD affects 1 in 54 children in the United States (US) and has higher prevalence in males than in females [2]. In the clinical community, there is an unmet need for molecular diagnostic biomarkers of ASD. MicroRNAs (miRNAs) have emerged as a class of small non-coding RNA molecules that are approximately 22 nucleotides in length and are highly conserved in eukaryotes [3,4,5]; miRNAs regulate gene expression by translational repression or mRNA degradation [4] and are emerging as promising biomarkers for diagnostic and therapeutic purposes in a number of disorders [6]. These molecules play a critical role in the regulation of cellular processes involving cell metabolism, differentiation, proliferation and cell death [7,8] and can control innate and adaptive immune pathways [9].

Significant differences in the ASD-associated miRNA expression profiles are found in a variety of target tissues (e.g., saliva, blood, serum and brain) [10,11], as miRNAs exhibit cell-specific and tissue-specific expression. Approximately 11% of copy number variant (CNV) loci found in individuals with ASD harbor miRNAs [12], and CNVs on chromosomes 1, 2 and 22 are associated with miRNA overexpression [13]. Some of the known ASD-associated genes are targets of miRNAs; for example, NRXN1 is targeted by miR-129, miR-181d, miR-381, miR-128, miR-23a, miR-27a, miR-539, miR-328 and miR-218 and SHANK3 is targeted by miR-15a, miR-484, miR-7, miR-128, miR-15b, miR-328 and miR-27a [14]. Thus, a greater understanding of the molecular mechanisms underlying this crosstalk between miRNA and gene expression is needed to develop biomarkers and therapeutic strategies for ASD.

To assess the potential role of miRNAs in ASD, in the present study, we performed a small RNAseq miRNA profiling in lymphoblastoid cell lines (LCLs) from ASD, and typically developing (TD) siblings and controls. As outlined below, in ASD LCLs, we found significant dysregulation of specific miRNAs, specifically miR-181 and miR-320a. The gene targets of dysregulated miRNAs in ASD were in the pathways of MAPK signaling, cytokine–cytokine receptor interaction, spliceosome, calcium signaling, WNT signaling and cancer.

## 2. Materials and Methods

### 2.1. Materials

RPMI 1640 culture media, penicillin/streptomycin, fetal bovine serum (FBS), phosphate buffered saline (PBS) and BCA Protein Assay Kit were all obtained from Thermo Fisher Scientific (Waltham, MA, USA). XF DMEM and XF-PS 96-well plates were obtained from Agilent Technologies (Santa Clara, CA, USA). The RNeasy mini kit was obtained from Qiagen (Hilden, Germany) and the High Capacity cDNA Reverse Transcription Kit and Power SYBR Green PCR Master Mix were procured from Applied Biosystems (Waltham, MA, USA). All other chemicals were obtained from Sigma-Aldrich (St. Louis, MO, USA).

### 2.2. Cell Lines and Culture

Ten pairs of LCLs derived from multiplex families with one male diagnosed with autistic disorder and one unaffected male sibling were obtained from the Autism Genetic Resource Exchange (AGRE; Los Angeles, CA, USA). Seven unrelated age-matched control LCLs derived from healthy male donors with no documented behavioral or neurological disorder or first-degree relative with a medical disorder that could involve abnormal mitochondrial function were obtained from Coriell Cell Repository (Camden, NJ, USA). Details of the LCLs are presented in Table 1. Age was not significantly different across groups as measured by the paired *t*-test. LCLs were maintained in RPMI 1640 culture medium with 15% FBS and 1% penicillin/streptomycin in a humidified incubator at 37 °C with 5% CO_2_. All ASD LCLs were linked to the results of the gold-standard Autism Diagnostic Observation Schedule (ADOS) assessments of the children from whom the LCLs were derived. These LCLs were used in a previous study examining bioenergetics [15].

### 2.3. Library Construction and Sequencing

Total RNA was extracted using Trizol reagent (Invitrogen, CA, USA) and sent to LC Sciences (Houston, TX, USA) on dry ice. RNA integrity and quality were determined using Bioanalyzer 2100 (Agilent, CA, USA) with RIN number > 7.0. Approximately 1 µg of total RNA was used to prepare a small RNA library according to the protocol of TruSeq Small RNA Sample Prep Kits (Illumina, San Diego, CA, USA). Single-end sequencing of 50 bp was performed on an Illumina Hiseq 4000 at LC Sciences following the vendor’s recommended protocol [16].

### 2.4. Bioinformatics Analysis

Raw reads were processed with ACGT101-miR (LC Sciences, Houston, TX, USA) to remove adapter dimers, junk, low-complexity reads, common RNA families (rRNA, tRNA, snRNA, and snoRNA) and repeats. Unique sequences with lengths of 18–26 nucleotides were mapped to specific-species precursors obtained from the miRBase 21.0 by a BLAST search performed to identify known miRNAs and novel 3p- and 5p-derived miRNAs. The remaining sequences were aligned against the miRbase (Release 21) (https://www.miRbase.org/) miRNA database, and perfectly matched sequences were considered to be conserved *Homo sapiens* miRNAs [16].

Normalization of sequence counts in each sample (or data set) was achieved by di-viding the counts by a library size parameter of the corresponding sample. The library size parameter is a median value of the ratio between the counts of a specific sample and a pseudo-reference sample. A count number in the pseudo-reference sample is the count of the geometric mean across all samples [16]. Differential expression of miRNAs based on normalized deep-sequencing counts was analyzed by selectively using Fisher’s exact test, chi-squared 2 × 2 test, chi-squared N × N test, Student’s *t*-test, or ANOVA based on the experiment design. The significance threshold was set to 0.01 and 0.05 in each test.

### 2.5. Target Prediction and Enrichment Analysis

To predict the genes targeted by most abundant miRNAs, two computational target prediction algorithms, TargetScan (http://www.targetscan.org/) and Miranda 3.3a (http://www.microrna.org/), were used to identify miRNA binding sites. Finally, the data predicted by both algorithms were combined and the overlaps were calculated. The GO terms and KEGG pathways of these most abundant miRNAs and miRNA targets were also annotated.

### 2.6. Quantitative Real-Time Polymerase Chain Reaction (qRT-PCR)

From LCLs, total RNA and microRNA were isolated using the miRNeasy Mini Kit (Qiagen, Valencia, CA, USA), and finally the RNeasy MinElute Cleanup Kit was used for miRNA isolation (Qiagen, Valencia, CA, USA) and cDNA was synthesized with the miScript II RT kit (Qiagen, Valencia, CA, USA). qRT-PCT was run in triplicate on a QuantStudio™ 6 Flex Real-Time PCR System (Thermo Fisher Scientific, Carlsbad, CA, USA). For miRNA expression analysis, cDNAs from cells were used in qRT-PCR reaction for miScript primer assays (Qiagen, Valencia, CA, USA). The miRNA primer assay ID and catalog number are shown in Supplementary Table S1A. For gene expression analysis, quantitative real-time polymerase chain reactions (qRT-PCRs) were performed using TaqMan Fast Master Mix (Thermo Fisher Scientific, Carlsbad, CA, USA), and details of the target gene and the assay ID are shown in Supplementary Table S1B. Taqman assays were duplexed with GAPDH/HPRT to normalize the mRNA expression.

For each primer assay, negative controls were run with water and cDNA samples. For no template controls (NTCs), a master mix with no cDNA was used. Data normalization was performed where appropriate with exogenous control (Ce-miR-39) and endogenous control (RNU6). Relative quantitation for miRNA and mRNA was calculated using the 2^−ΔΔC*t*^ method.

### 2.7. Statistical Analysis

All experimental data are presented as means ± SEM (standard error of the mean), and the differences between the two groups were examined using Student’s *t*-test (2-tailed). The limit of significance accepted for all statistical analyses was *p*-value less than 0.05.

## 3. Results

### 3.1. Identification of Differentially Expressed miRNAs

Pie chart distribution annotation for the total vs. unique distribution of small RNAs in ASD and control samples shows substantial difference (Supplementary Figure S1A,B). The length distribution of the validated reads was counted in ASD, siblings, and control groups, and the lengths of the validated reads centered on 22 nt. The lengths of most validated reads concentrated between 21 and 24 nt (Supplementary Figure S2A). The calculation of the length of unique reads at 22 nt was higher than that at other lengths in ASD, siblings, and control groups (Supplementary Figure S2B).

**Figure 1:**
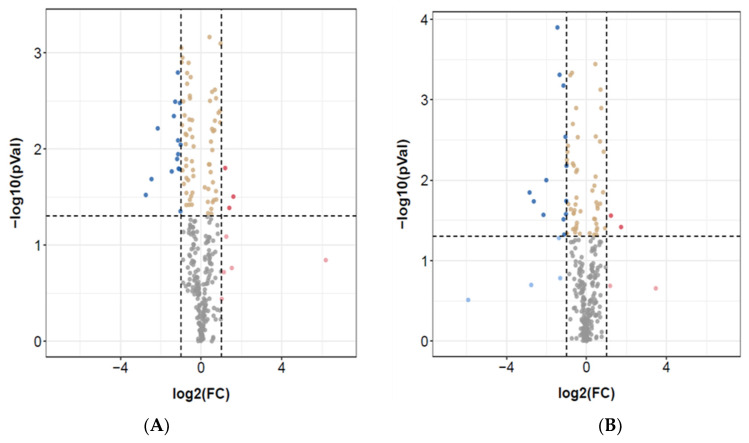
Differential expression of miRNAs in LCL groups. (**A**) Volcano plot of differentially expressed miRNAs in ASD and control LCLs. (**B**) Volcano plot of differentially expressed miRNAs in ASD and Siblings LCLs. The red dots are up-regulated, and the blue dots are down-regulated; the gray dots denote no change; the yellow dots are not significant. *n* = 9–10 in each group. To determine significant genes (red and blue color dots), the *p*-value cut-off was set to 0.05.

Differential expression analysis, as shown in the volcano plot, identified significant known and novel miRNAs (Figure 1) using |log2 fold change| and *p* ≤ 0.05 criteria. For example, the ASD vs. control group showed 15 down-regulated and 3 up-regulated miRNAs out of 269 detected (Figure 1A), whereas in the ASD vs. Siblings group, there were 2 up-regulated and 13 down-regulated miRNAs out of 267 detected (Figure 1B).

**Figure 2:**
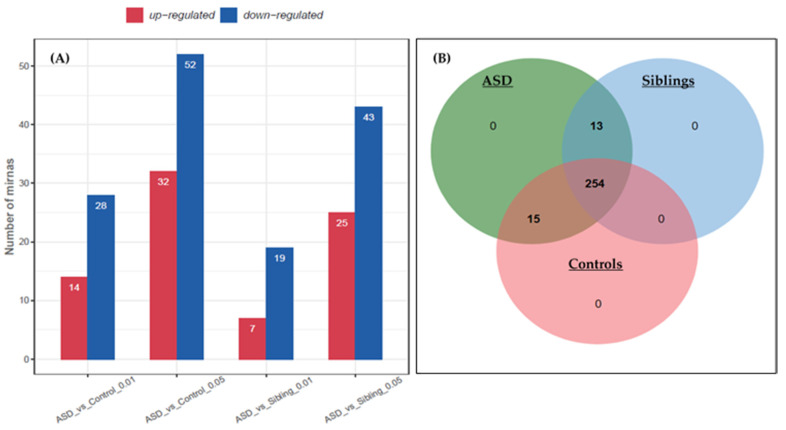
Differential expression of miRNAs in ASD and control LCLs as well as in ASD and siblings. (**A**) Total number of miRNA expression at *p* < 0.01 and *p* < 0.05. (**B**) Venn diagram of all the dysregulated miRNAs in the groups, showing miRNAs which are unique and common in the three groups.

A total of 84 differentially expressed miRNAs were identified in the ASD vs. control groups, out of which 32 were up-regulated and 52 down-regulated (a cut-off at *p* < 0.05), and ASD vs. siblings gave a total of 68 differentially expressed miRNAs, out of which 25 were up-regulated and 43 were down-regulated (Figure 2A), respectively. Moreover, the number of significantly expressed miRNAs fell when criteria were made stringent (a cut-off at *p* < 0.01, Figure 2A). To investigate the potential function and gene expression of the known and novel miRNAs, a Venn diagram of differentially expressed miRNAs in the ASD, siblings, and control groups shows that a total of 254 miRNAs were common in the three groups, with 13 common in ASD vs. siblings, and 15 common in ASD vs. controls, respectively (Figure 2B).

The list of top miRNAs (Table 2) shows fold change (down-regulated and up-regulated) along with their *p*-values for the ASD and control as well as ASD and siblings groups, respectively. Supplementary Figure S3A,B shows the results of Heatmap, where the colors show the fold change value with a two-color gradient for expression levels, from red (up-regulated) to green (down-regulated), in ASD vs. control and ASD vs. siblings, respectively. Heatmaps show that the distribution of miRNAs was very distinct for miR-21, Let-7a, miR-26a, and miR-146b, as it differentiated ASD from unrelated controls only (Supplementary Figure S3A). On the other hand, hsa-miR-320a differentiated ASD vs. siblings (Supplementary Figure S3B). These results demonstrated that miRNAs were differentially expressed in ASD vs. control and ASD vs. siblings, respectively.

### 3.2. qPCR Validation of Differentially Expressed miRNAs

**Figure 3:**
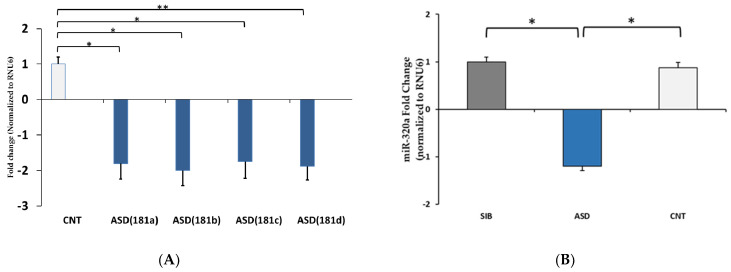
qPCR validation of miRNA expression: (**A**) miR-181a and other miR-181 family members (181b-5p, miR-181c-5p, and miR-181d-5p) and (**B**) miR-320a in LCLs. SIB = siblings; CNT = controls; ASD = autism spectrum disorder; data are means ± SEM of *n* = 9–10 samples in each category. Error bars represent standard error of the mean. miRNA expression was normalized to RNU6 for each group. * *p* ≤ 0.05.; ** *p* ≤ 0.01.

To validate the sequencing results of miRNA expression, we selected two miRNAs, hsa-miR-181a-5p from ASD vs. controls and hsa-miR-320a from ASD vs. siblings (Table 2), for quantitative real-time polymerase chain reaction (qPCR) analysis. The expression level of hsa-miR-181a-5p shows significant down-regulation (*p* < 0.05) in the ASD group compared to the control group (Figure 3A). We also examined other members of the miR-181 family (b, c, and d), which were also found to be down-regulated in the ASD group (Figure 3A). The expression of miR-320a was also significantly (*p* < 0.05) down-regulated in the ASD vs. siblings group (Figure 3B). The qPCR confirmed the RNAseq results, indicating that the expression patterns of these selected miRNAs were similar to the sequencing results.

### 3.3. Pathway Analysis

**Figure 4:**
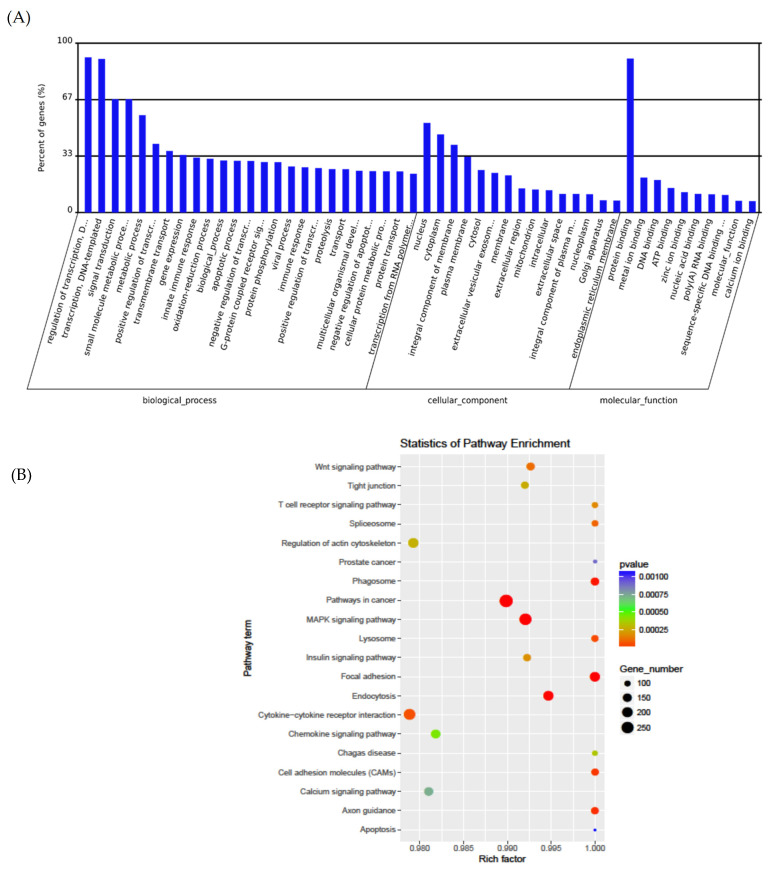
Enrichment analysis of the differentially expressed miRNAs. (**A**) GO function classification of the target genes of differentially expressed miRNAs. The top 25, 15, and 10 GO terms in biological processes, cellular components, and molecular functions, respectively. (**B**) The top 20 enriched KEGG signaling pathways of the target genes of differentially expressed miRNAs.

Predicting the target genes of miRNAs that may be involved in translation inhibition in ASD is important. GO analysis indicated terms in biological processes, cellular components, and molecular functions, which were significantly enriched for these target genes (Figure 4A). The most significantly enriched GO terms included the regulation of transcription, signal transduction, protein binding, ATP binding, nucleus, cytoplasm, and innate immune response (Figure 4A). The graph in Figure 4B represents the target genes of the differentially dysregulated miRNAs enriched in GO terms, where the color of the circle indicates the statistical significance expressed in log10 values and the size of the circle indicates the number of target genes involved. KEGG pathway analysis showed that the target genes were notably enriched in the pathways involving MAPK signaling, cytokine–cytokine receptor interaction, spliceosome, calcium signaling, WNT signaling, and cancer (Figure 4B).

**Figure 5:**
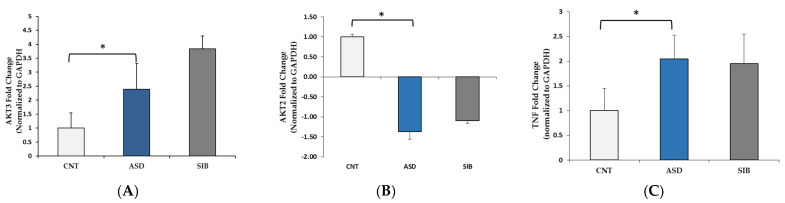
Gene expression of (**A**) AKT serine/threonine kinase 3 (AKT3), (**B**) AKT serine/threonine kinase 2 (AKT2), and (**C**) tumor necrosis factor α (TNF) in ASD, controls, and siblings. CNT = controls, ASD = autism spectrum disorder, and SIB = siblings. Data are means ± SEM of *n* = 9–10 samples in each category. Error bars represent standard error of the mean. Gene expression was normalized to GAPDH for each group. * *p* ≤ 0.05.

From the KEGG analysis (Table 3), the following genes were selected for the validation in LCLs: AKT2, AKT3, ATM, CAMK2A, EIF4E2, GZMB, IL2, IL17A, PIK3CG, SCN2A, and TNFα (Supplementary Table S1A). We observed no expression levels of CamK2A, PIK3CG, EIF4E2, ATM, GZMB, and IL-2. Figure 5 shows gene expression levels in the ASD group vs. control group as well as ASD vs. siblings. AKT3 and TNFα expressions were up-regulated in the ASD group compared to controls (Figure 5A,B); AKT2 expression was significantly down-regulated in the ASD group compared to controls (Figure 5A–C). The expression levels of SCN2A in the control and ASD groups were not significantly different.

**Figure 6:**
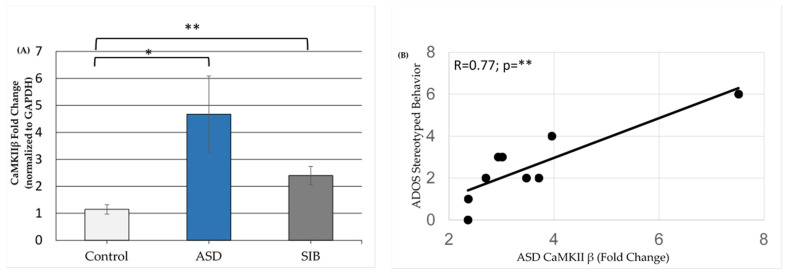
(**A**) CaMKII β expression in LCLs. (**B**) Correlation of CaMKII β expression and stereotyped behavior per ADOS criteria. CNT = control, ASD = autism spectrum disorder, and SIB = Siblings; *n* = 9–10 in each group; * *p* ≤ 0.05.; ** *p* ≤ 0.01.

Though we were unable to detect expression of CaMKII α subunit in the LCLs, the expression of the other isoform CaMKII β was detectable in LCLs. ASD LCLs demonstrated to be a significantly higher expression of CaMKII β compared to the non-related control LCLs (Figure 6A). However, the sibling controls demonstrated an intermediate increase in expression between the non-related controls and the ASD LCLs. We also examined the relationship between the expression of CAMK2B and scores on the components of the ADOS for the ASD participants. As seen in Figure 6B, the index of stereotyped behavior and restricted interest score demonstrated a positive correlation with CaMKII β expression (R = 0.77, *p* < 0.01).

## 4. Discussion

A number of large-scale genome-wide association studies (GWAS), as well as whole exome sequencing (WES) and whole genome sequencing (WGS), show the complex genetic underpinnings of ASD [17,18,19]. In this study, we screened LCLs from children with ASD using small RNAseq analysis and identified a few top miRNAs (Table 2). This is only one of the few studies on miRNA dysregulation in ASD [20], and only three studies have specifically used LCLs [21,22,23]. Talebizadeh et al. [22] observed up-regulation of miR-23a/b, miR-132, miR-146a/b, and miR-663 and down-regulation of miR-92a-1, miR-92a-2, miR-320, and miR-262 in 6 cases and 6 controls. Ghahramani Seno et al. [21] identified the up-regulation of miR-196a, miR-650, miR-338-3p, and miR-125b using a microarray in 20 cases and 22 controls. Sarachana et al. [23] showed the down-regulation of miR-182, miR-136, miR-518a, miR-153, and miR-211 and up-regulation of miR-185, miR-103, miR-107, miR-29b, miR-194, miR-524, and miR-191 in 5 cases and 9 controls. Though these studies used small sample sizes and showed variable results, they showed that the miRNAs are dysregulated in ASD.

Other larger studies on other tissues have also provided interesting findings. Another study using postmortem cerebellar cortex tissue showed down-regulation of miR-15a and miR-21 and up-regulation of miR-181d and miR-320a in 13 cases and 13 controls [14]. Vasu et al., who conducted perhaps the largest miRNA study in ASD, found down-regulation of miR-151a-3p, miR-181b-5p, miR-320a, miR-328, miR-433, miR-489, miR-572, and miR-663a and up-regulation of miR-101-3p, miR-106b-5p, miR-130a-3p, miR-195-5p, and miR-19b-3p in the serum of 55 individuals with ASD and 55 age- and gender-matched controls [24].

In our study, KEGG enrichment analysis showed that these miRNAs target genes in signaling pathways are important in ASD. We concentrated on two selected miRNAs, hsa-miR-181a-5p and hsa-miR-320a, from the top candidate list since they are highly expressed in the brain and spinal cord [25] and have been identified as dysregulated in previous ASD studies [14,22,24].

In the current study, the levels of all the miR-181 family members were significantly down-regulated in the ASD group (Figure 3A). The miR-181 family is evolutionarily conserved and strongly expressed in the brain and has extensive roles in development, neurodegeneration, and cancer [26]. Cerebral ischemic injury is associated with overexpression of miR-181a [27,28,29], and inhibition of miR-181 expression during cerebral ischemic injury can be neuroprotective [30]. In fact, polymorphisms in miR-181b may modulate risk of cerebral ischemic injury in humans [27] and may also be involved in cardiac ischemia [31]. In astrocytes, miR-181 regulates mitochondrial function and apoptosis [32], and neuroinflammation [33] and has a role in ischemia neuroprotection [27]. A critical role of miR-181 was postulated in synaptic plasticity [34]. These authors showed that alteration of miR-181 levels contributes to the cognitive neuropathological development in a 3xTg-ASD mouse model and that overexpression of miR-181 in SH-SY5Y cells reduces SIRT-1 and c-Fos protein expression.

miR-181 family members are also involved in metabolic regulation in the immune system [35]. Chen et al. [36] suggested that miR-181 is a positive regulator of B cell differentiation and that its expression acts independently for differentiation into B cells (CD19+) and cytotoxic T cells (CD8+). miR-181 may be involved in post-traumatic immune response through the modulation of TNFα [37] and differentiation of NK cells [38], two abnormalities repeatedly associated with ASD [39]. The ATG5 gene is a target for miR-181a and has been described as a new autophagy-regulating miRNA as overexpression of miR-181a resulted in the attenuation of starvation- and rapamycin-induced autophagy in MCF-7, Huh-7, and K562 cells [40].

The miR-320 family is weakly expressed in the ischemic heart, protects against I/R-induced cardiomyocyte death and apoptosis [41], and has been shown to act as a tumor suppressor in different cancer types [42].

Both miR-181a-5p [43,44] and miR-320a [45] target AKT3, a key regulator of the PI3K-AKT-mTOR signaling pathway, a pathway that is known to be dysregulated in ASD and is a potential therapeutic target in ASD [46]. miR-320a has been shown to promote myocardial fibroblast proliferation by regulating the PIK3CA/Akt/mTOR signaling pathway in HEH2 cells [47]. Bone marrow mesenchymal stem cells alleviate severe acute pancreatitis and reduce inflammatory responses and apoptosis by secreting miR-181a-5p to target the PTEN/Akt/TGF-β1 signaling pathway [16].

CaMKII also has a critical role in neuronal function in the post-synaptic neuron, which is closely associated with memory and learning, primarily through the modulation of long-term potentiation and NMDA-dependent synaptic plasticity [48,49]. CaMKII has an important role in the regulation of NMDA receptors at the post-synaptic density, which has significant implications for neuronal excitability through glutamate transmission [50]. This may be significant in neuropsychiatric disorders, as dysregulation of long-term potentiation is being recognized in syndromes, which include the Gilles de la Tourette syndrome [51], and disrupted glutamate transmission is being recognized as a treatment target in obsessive-compulsive disorder [52]. Furthermore, CaMKII is being recognized to have an important role in cognition and learning as mutations in CaMKIIα and CaMKIIβ are associated with intellectual disability [53] and ASD-related behaviors such as hyperactivity, social interaction deficits, and repetitive behaviors [54].

Although this study has provided some interesting findings, there are certainly limitations. The most notable limitation is the sample size. Thus, future studies should verify these findings with larger sample sizes. In addition, this study was conducted on immortalized cell lines, not fresh tissue. Although the clinical characteristics of the ASD participants obtained at the time of tissue collection did correlate with gene expression in the immortalized cell lines, the symptoms wax and wane and sometimes improve in individuals with ASD. Thus, examining the changes in gene expression over time and their correlation with ASD symptoms would validate our findings. Nevertheless, this study provides an insight into promising novel biomarkers, which may provide an insight into the dysfunctional cellular regulatory mechanisms associated with ASD.

## 5. Conclusions

Taken together, our study shows that dysregulation of miRNAs in ASD LCLs impacts multiple signaling pathways. KEGG enrichment analysis showed dysregulated miRNAs, which target genes in the pathways involving MAPK signaling, spliceosome, calcium signaling, and WNT signaling, among others. Other members of the miR-181 and miR-320 family have closely related sequence homology and may have shared gene targets in the etiology of ASD, as they can target several immune genes, such as TNFα, AKT3, and AKT2. The miR-181 family is related to multiple physiological systems and mechanisms known to be dysregulated in ASD, including synaptic plasticity [34], mitochondrial metabolism [32], neuroinflammation [33], immune system regulation [35], NK cell differentiation [38], TNFα modulation [37], and regulation of the mTOR [40,43,44] and PTEN [16] signaling pathways. We linked ASD symptomology to changes in miRNA dysregulation through the modulation of CaMKII, a central cellular regulator associated with a wide range of ASD-associated characteristics, including abnormalities in learning, repetitive behaviors, and social interactions [52,53,54]. Although this is one mechanism that links the findings of this study to clinical symptoms, given the multiple pathways regulated by the miR-181 family, many other aforementioned mechanisms contribute to ASD symptoms through downstream effects from dysregulation of the miR-181 family. Our work adds to the list of miRNAs that pair to specific mRNAs to silence gene expression, which may be critical in ASD and demonstrates how miRNAs can affect the multiple physiological systems associated with ASD.

## 6. Patents

There are no patents to declare.

## Figures and Tables

**Table 1:** Lymphoblastoid cell lines (LCLs) used in this study. LCLs are from children with autistic disorder and typically developing siblings as well as unrelated age- and gender-matched control. The average age of each group is provided. There is no significant difference in age across groups.

Unrelated Controls		Sibling Controls		Autistic Disorder	
ID	Age	ID	Age	ID	Age
GM09659	4	AU1393305	4	AU1393306	3
GM11599	9	AU038803	9	AU038804	8
GM15862	11	AU0939302	8	AU0939303	11
GM10153	10	AU1267303	7	AU1267302	10
GM16007	12	AU1348302	13	AU1348303	12
GM09621	8	AU1344303	4	AU1344302	7
		AU1280304	1	AU1280302	7
		AU1215305	2	AU1215301	12
		AU008405	9	AU008404	13
GM11626	13	AU1165303	12	AU1165302	13
Average (SD)	9.6 (3.0)		6.9 (4.1)		9.6 (3.3)

**Table 2:** Top differentially expressed miRNAs in the ASD vs. control groups and ASD vs. siblings.

ASD vs. Controls			ASD vs. Sibling		
miRNA ID	log2(FC)	−log10(pVal)	miRNA ID	log2(FC)	−log10(pVal)
PC-5p-35875_59	−2.7	1.521688009	hsa-miR-451a_R-1	−5.9	0.5123528
hsa-mir-18b-p3	−2.5	1.686252607	PC-5p-8577_335	−2.8	1.8479091
hsa-miR-451a_R-1	−2.5	2.213487364	hsa-miR-96-5p_R-2	−2.7	0.6983189
hsa-miR-92a-2-5p_R+1	−1.4	1.766163822	hsa-miR-4485-3p_L+3R+2	−2.6	1.7373332
PC-3p-16340_153	−1.3	2.341434396	hsa-miR-99b-5p	−2.2	1.5697133
hsa-let-7i-3p_R-2	−1.3	2.490787931	hsa-miR-4521_R+3	−1.5	3.8991403
hsa-miR-4437_L+2	−1.2	1.895455942	hsa-let-7e-5p	−1.4	1.2824785
hsa-mir-5100-p3_1ss17TC	−1.1	2.794312062	hsa-miR-125a-5p_R-1	−1.3	0.7832214
PC-3p-12325_216	−1.1	2.088661411	hsa-miR-766-5p_R-1	−1.2	3.1763432
hsa-miR-181a-5p	−1.1	1.945138848	hsa-miR-1270	−1.1	1.5145443
hsa-miR-363-3p_R+1	−1.1	1.795244892	hsa-miR-106a-5p	−1.1	1.3240308
hsa-miR-10a-5p_R-1	−1.1	1.790370904	hsa-miR-320a	−1.0	2.5399924
hsa-miR-20b-5p	−1.0	1.352069587	hsa-miR-1246_L-1R+1	−1.0	1.5790633
hsa-miR-1271-5p	1.2	1.801259553	hsa-miR-5701_1ss2TG	1.2	1.560449
hsa-miR-151a-5p	1.6	1.504469843	hsa-miR-150-5p	1.7	1.4184257

**Table 3:** Significantly enriched KEGG pathways in differentially expressed miRNAs in the dataset..

Pathway	*p*-Value	Genes Involved
**Up-regulated Pathways**		
PI3K-Akt signaling pathway	0.002	SGK3, OSMR, FGF14, COL3A1, HGF, C8ORF44-SGK3, ATF2, LAMA2, VEGFA, SOS2, PIK3AP1, EIF4E2, AKT2
ErbB signaling pathway	0.006	EREG, ERBB4, SOS2, MAPK9, CAMK2A, AKT2
Neurotrophin signaling pathway	0.02	RPS6KA6, SOS2, MAPK9, CAMK2A, PRKCD, AKT2
HIF-1 signaling pathway	0.04	PFKFB3, VEGFA, CAMK2A, EIF4E2, AKT2
Adrenergic signaling: cardiomyocytes	0.05	ACTC1, ADRB1, ATP1A2, CAMK2A, AKT2, ATF2
**Down-regulated Pathways**		
Transcriptional misregulation in cancer	<0.0001	SUPT3H, FLT1, UTY, GZMB, AFF1, ATM, ATF1, MLF1, CCR7, PBX1, ETV6, PBX3, MLLT3, HIST1H3I
T cell receptor signaling pathway	<0.01	PIK3CG, TNFα, RASGRP1, NFATC2, NFATC3, AKT3, TEC, IL2
Chagas disease (American trypanosomiasis)	<0.01	PIK3CG, TNFα, GNAI1, FADD, TRAF6, PLCB1, AKT3, IL2
Sphingolipid signaling pathway	<.015	PIK3CG, TNFα, SPTLC1, GNAI1, ACER2, PLCB1, AKT3, ASAH2
PI3K-Akt signaling pathway	0.018	PIK3CG, FLT1, KITLG, RPS6KB1, TCL1B, COL4A6, HSP90B1, YWHAH, PRLR, ITGA5, COL6A2, THBS3, AKT3, SPP1, IL2
Inflammatory mediator regulation of TRP channels	0.02	PIK3CG, HRH1, ADCY2, CALM3, PLA2G6, PLCB1, ALOX12
Estrogen signaling pathway	0.02	PIK3CG, HSP90B1, ADCY2, GNAI1, CALM3, PLCB1, AKT3
Glucagon signaling pathway	0.02	GCG, ADCY2, ACACA, CALM3, PLCB1, AKT3, PYGB
cGMP-PKG signaling pathway	0.03	PIK3CG, ADCY2, GNAI1, CALM3, NFATC2, PLCB1, NFATC3, AKT3, VDAC1
Cytokine–cytokine receptor interaction	0.03	IL17A, TNFRSF11B, CCR7, TNFα, FLT1, PRLR, CXCL16, IL25, KITLG, BMPR1A, IL2
Graft-versus-host disease	0.035	TNFα, GZMB, HLA-DOA, IL2
Apoptosis	0.04	PIK3CG, TNFα, FADD, AKT3, ATM
Allograft rejection	<0.05	TNFα, GZMB, HLA-DOA, IL2
HTLV-I infection	0.05	PIK3CG, TNFα, ADCY2, HLA-DOA, NFATC2, NFATC3, AKT3, ATF1, ATM, VDAC1, IL2

## Data Availability

Data is available upon request.

## Supplementary Materials

The following are available online at https://www.mdpi.com/article/10.3390/jpm11090922/s1: Figure S1: Distribution of small RNA type; Figure S2: Length distribution of miRNAs; Figure S3A: Heatmap for ASD vs. unrelated controls; Figure S3B: Heat map for ASD vs. Siblings; Table S1: miRNA and gene expression assays for qRT-PCR.
