# Supplementary material for: MicroRNA Expression Profiles in Autism Spectrum Disorder: Role for miR-181 in Immunomodulation
Source: J Pers Med. 2021 Sep 17;11(9):922. doi: 10.3390/jpm11090922 (PMC8469245; doi:10.3390/jpm11090922)
Supplement: Supplementary file 1 [file jpm-11-00922-s001.zip › Supplementary/Supplementary Figure S1.pdf]

A)

B)

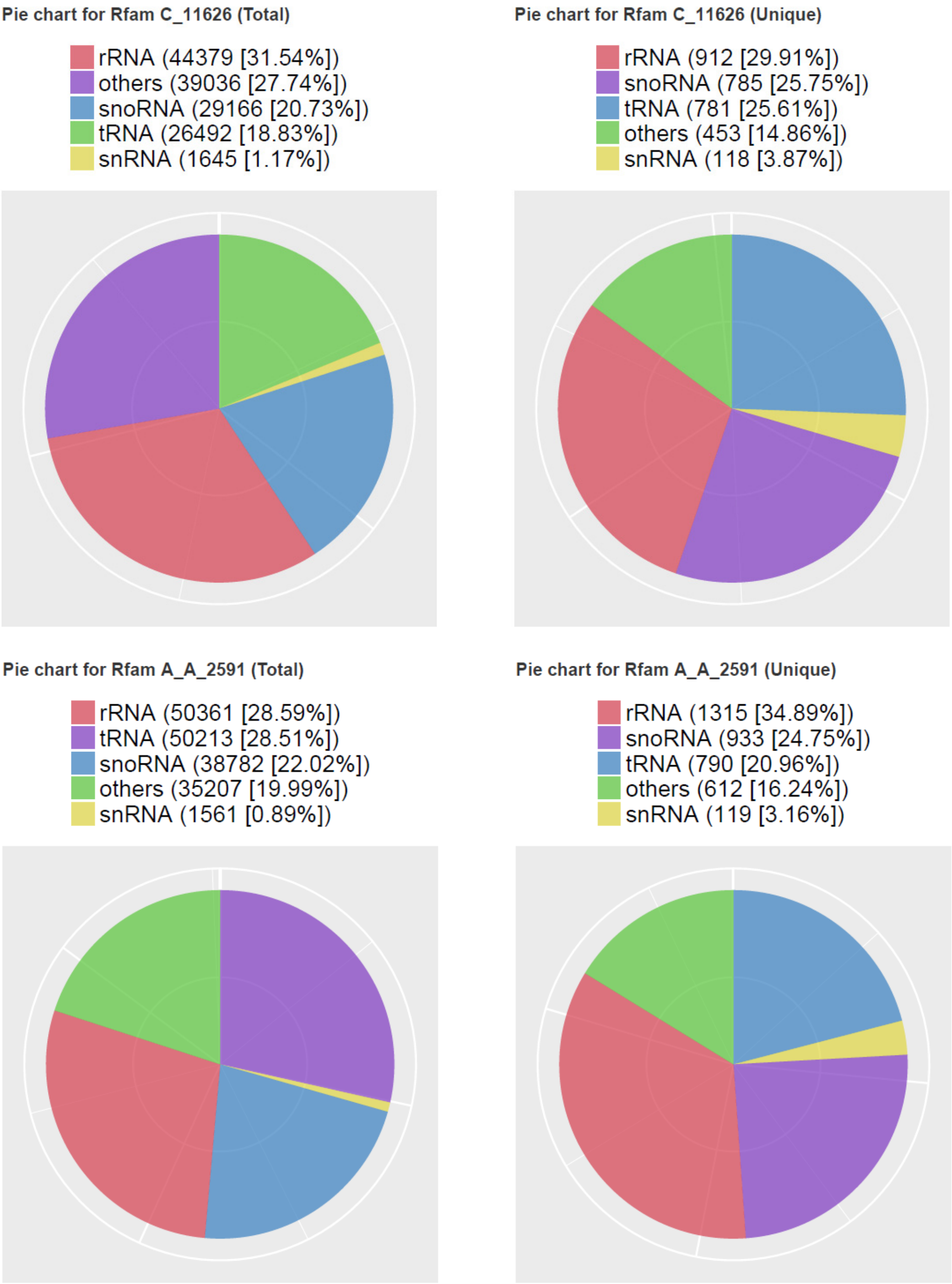

Supplementary Figure S1: Pie chart distribution annotation of small RNAs total vs unique shown as an example in ASD (2591) and control (11626) sample.
