# Supplementary material for: MicroRNA Expression Profiles in Autism Spectrum Disorder: Role for miR-181 in Immunomodulation
Source: J Pers Med. 2021 Sep 17;11(9):922. doi: 10.3390/jpm11090922 (PMC8469245; doi:10.3390/jpm11090922)
Supplement: Supplementary file 1 [file jpm-11-00922-s001.zip › Supplementary/Supplementary Table S1.pdf]

Supplementary Table S1: miRNA and gene expression assays for qRT-PCR

A) Primer miScript Assays (Qiagen, Valencia, CA)

SYBR Green Assays	Catalog number	Entrez Gene ID
Hsa-miR-320a	MS00014707	407037
Hsa-miR-181a-5p	MS00008827	406954
Hsa-miR-181b-5p	MS00006699	406955
Hsa-miR-181c-5p	MS00008841	406957
Hsa-miR-181d-5p	MS00031500	574457
Ce_miR39_1	MS00019789	N/A
Hs_RNU6	MS00033740	N/A

B) TaqMan gene expression assays (ThermoFisher Scientific, Carlsbad, CA)

Taqman Assays	Assay ID	Entrez Gene ID
AKT3	Hs00987343_m1	10000
TNF	Hs00174128_m1	7124
AKT2	Hs01086099_m1	208
SCN2A	Hs01109871_m1	6326
ATM	Hs00175892_m1	472
GZMB	Hs00188051_m1	3002
CAMK2A	Hs00947041_m1	815
PIK3CG	Hs00932391_m1	5294
EIF4E2	Hs00601369_g1	9470
IL2	Hs00174114_m1	3558
CAMK2B	Hs00365799_m1	816
GAPDH	Hs02758991_g1	2597
