# Supplementary figures and images for: MicroRNA Expression Profiles in Autism Spectrum Disorder: Role for miR-181 in Immunomodulation
Source: J Pers Med. 2021 Sep 17;11(9):922. doi: 10.3390/jpm11090922 (PMC8469245; doi:10.3390/jpm11090922)

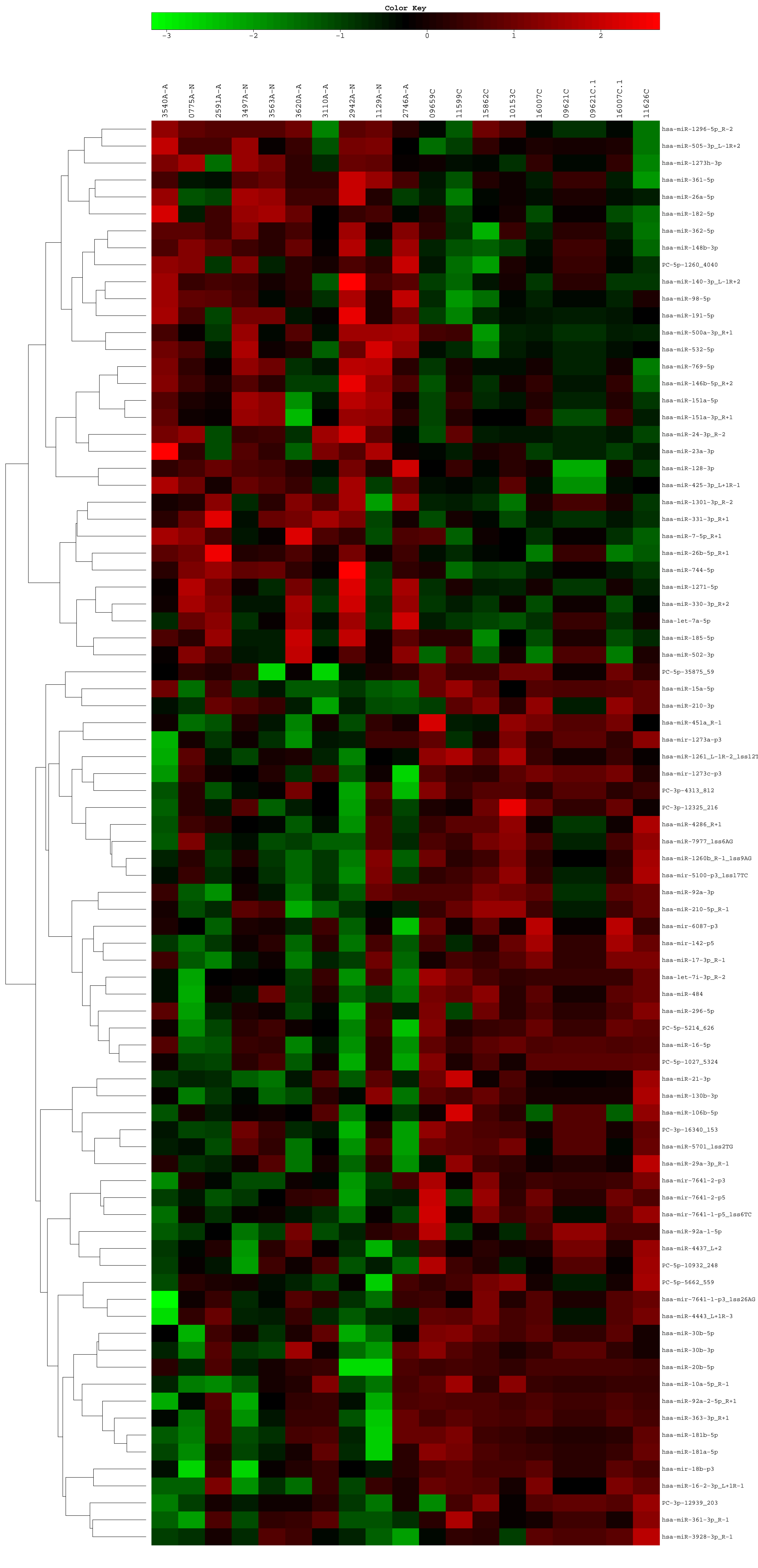

Supplement: Supplementary file 1 [file jpm-11-00922-s001.zip › Supplementary/Supplementary Figure S3A.pdf]

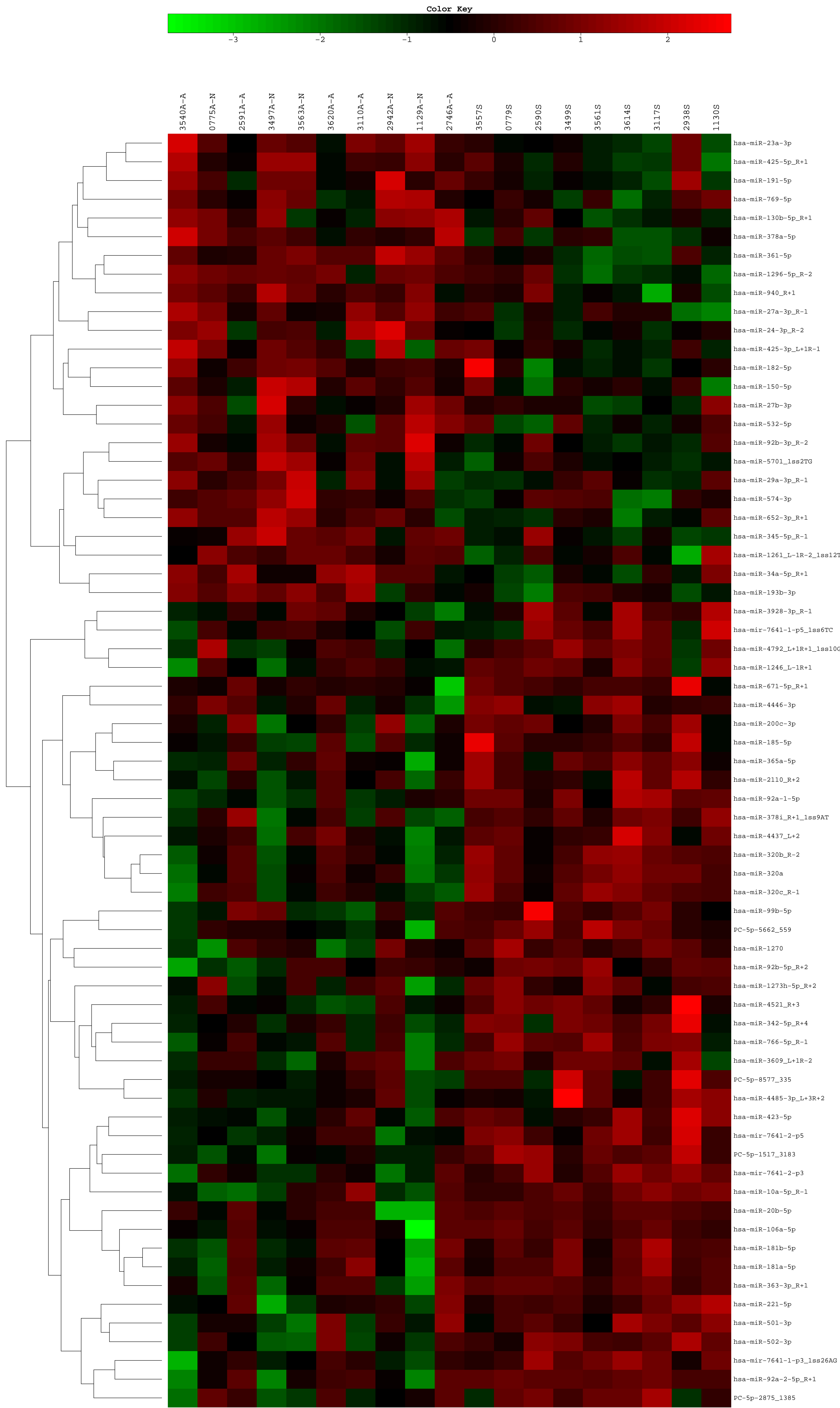

Supplement: Supplementary file 1 [file jpm-11-00922-s001.zip › Supplementary/Supplementary Figure S3B.pdf]
